# Prerequisites for ethical leadership in health and social care: Integrative review

**DOI:** 10.1177/09697330251366593

**Published:** 2025-09-22

**Authors:** Anniina Seere, Riitta Suhonen, Johanna Wiisak

**Affiliations:** 8058 University of Turku; 8058 University of Turku; Turku University Hospital; Wellbeing Services County of Southwest Finland; Lero – the Science Foundation Ireland Research Centre for Software; University of Galway; 8058 University of Turku

**Keywords:** Ethical leadership, healthcare, inductive content analysis, integrative review, organizational ethics, prerequisites, social care

## Abstract

Health and social care organizations face structural reforms, workforce shortages, and increasing ethical demands. These pressures underscore the importance of ethical leadership, particularly from leaders managing complex services. While ethical leadership improves integrity, trust, and wellbeing, limited research has examined the prerequisites enabling its realization, especially from the perspectives of leaders in health and social care settings. Therefore, this integrative literature review aimed to identify and synthesize the prerequisites for ethical leadership in these contexts from a leadership perspective. The review was carried out following the PRISMA guidelines, with the protocol registered in PROSPERO and quality assessed using the Mixed Methods Appraisal Tool. A systematic search across six databases resulted in nine peer-reviewed studies (2010–2025). Inductive content analysis identified five categories of prerequisites for ethical leadership: (1) leader-centric prerequisites including ethical sensitivity and moral courage; (2) ethical organizational culture based on shared values; (3) leadership support such as mentoring and development; (4) ethical guidelines supporting consistent decision-making; and (5) resource sufficiency to enable ethical action. Ethical leadership emerged as both individual competencies and a dynamic process embedded in organizational structures. The ethical capacity of leaders was found to depend on personal capabilities and structural support. These findings inform leadership development and organizational strategies aimed at strengthening ethical practices in complex care environments.

## Introduction

Ethical leadership, grounded in honesty, fairness, care, and respect, forms the foundation of responsible decision-making.^1^ Ethical leadership is conceptualized not as a personal moral judgment of the leader but rather as a set of skills and practices that can be learned, developed, and supported through organizational structures and culture. Ethical leaders serve as moral role models who foster a culture in which ethical considerations guide decision-making at all organizational levels.^2,3^ In health and social care organizations where leaders frequently face ethically complex and high-stakes situations, ethical leadership contributes to client- and patient-centered care, upholding professional integrity and supporting staff wellbeing, motivation, and trust.^4,5^

Despite growing interest in ethical leadership across sectors, there is still limited knowledge about the specific prerequisites required for its successful implementation in social and healthcare contexts. Although ethical leadership has been increasingly studied,^2–4^ existing research has primarily focused on outcomes such as job performance, job satisfaction, or service behaviors—most often from the perspective of employees, particularly nurses.^6,7^ Less is known about what social care and healthcare leaders themselves perceive as necessary for ethical leadership to emerge and be sustained in practice. This gap is particularly important in light of the growing complexity and demands facing health and social care organizations.^8^

Recent studies suggest that ethical leadership is not solely grounded in individual moral competencies, but that it also depends on structured and sustainable organizational enablers such as leadership support, trust, shared accountability, and an ethical climate.^2,3^ These findings underscore that ethical leadership requires more than personal values; it also relies on organizational prerequisites that enable and reinforce ethical conduct. Without a clear understanding of these prerequisites, ethical leadership may become inconsistent, reactive, or overly dependent on individual leaders, rather than embedded in supportive organizational structures, cultures, and resources.

This integrative literature review aims to identify and synthesize the prerequisites for ethical leadership in health and social care, as described in empirical studies from the perspective of organizational leaders. By clarifying these prerequisites, whether individual, organizational, or structural, the review seeks to inform leadership development and organizational strategies and policies that foster sustainable and ethically grounded leadership practices across all levels of governance.

## Background

Structural changes in the health and social care sectors, such as leadership restructuring, staff shortages, and growing ethical demands, have intensified pressure on leaders, particularly those overseeing large professional groups like nurses, the largest workforce segment in these sectors. The reduction of managerial positions has placed increased demands on the scope and clarity of leadership roles.^9^ These pressures, combined with heavier workloads and fragmented service delivery, put strain on leaders in health and social care, potentially undermining their ability to maintain ethical leadership. Such conditions can directly influence the quality of care and the overall resilience of health and social care organizations.^10^ Ethical leadership in this context is also shaped by organizational ethics, that is, the shared norms, responsibilities, and structures that support morally sound practices and decision-making beyond the individual level.^11^ As ethical leadership fosters trust, job satisfaction, and improved institutional outcomes, supporting it is not only beneficial but a key factor in building ethically sustainable health and social care systems.^4^

While ethical leadership has been widely studied in corporate settings, the specific features of health and social care organizations require a context-sensitive examination of the conditions that support its implementation. This sector is uniquely characterized by constant value-laden decisions, emotional labor, and direct human impact, which may amplify ethical tensions compared to many other professional contexts.^4,6^ To conceptually frame these conditions, this review draws on selected perspectives from ethical leadership theory, transformational leadership, and servant leadership. These theoretical approaches help to illustrate how prerequisites for ethical leadership can be understood at multiple levels: as individual characteristics (e.g., integrity and fairness),^1^ organizational structures (e.g., shared values and aligned communication),^1,12,13^ and cultural elements (e.g., care orientation and staff wellbeing).^12,13^ Although these theories are not applied as analytical frameworks in this review, they provide a conceptual foundation for understanding the multifaceted nature of ethical leadership prerequisites.

In this review, the term *prerequisites* refers to key conditions that enable or support ethical leadership in health and social care organizations. While the term may suggest fixed or strictly necessary conditions, we use it here to more flexibly refer to factors that typically support or enable ethical leadership in context. In line with Scharf and Berntson,^8^ who define managerial prerequisites as situational combinations of demands and resources, we understand prerequisites as individual and organizational factors that ideally foster ethical leadership, even if they are not always fully present in practice.

Previous reviews have examined ethical leadership from various perspectives. Singh and Vashist^3^ focused on its effects on organizational culture, staff wellbeing, and patient safety in health and social care, while Wu et al.^14^ analyzed ethical leadership as part of broader leadership styles, particularly its connection to job satisfaction and organizational citizenship. From a diversity, equity, and inclusion perspective, Coleman and Taylor^15^ emphasized that integrating diversity, equity, and inclusion principles into leadership may enhance ethical leadership and organizational effectiveness. Brennan and Monson^16^ explored the relationship between professionalism and ethics, highlighting how organizations can foster ethical practices through structural and cultural means. Suhonen et al.^11^ reviewed organizational ethics in healthcare, stressing the importance of clear ethical structures and leadership support.

While these reviews confirm the relevance of ethical leadership to various organizational outcomes, there has not yet been a review conducted that has systematically examined the prerequisites of ethical leadership, particularly from the leader’s perspective. To fill this notable gap in literature, by identifying the elements that support ethical leadership and analyzing the contextual factors influencing its implementation, this review contributes to developing targeted leadership programs and supportive organizational structures. Knowledge obtained from this review contributes to a clearer understanding of the prerequisites for ethical leadership in health and social care. These insights can support leadership development, policy-making, and organizational strategies aimed at fostering sustainable and ethically grounded leadership in complex care environments.

## Review

### Aim

The objective of this integrative literature review is to systematically identify and synthesize existing empirical studies on the prerequisites for ethical leadership in health and social care, as described from the perspective of organizational leaders. This review seeks to answer the research question: *What are the prerequisites for ethical leadership in health and social care?*

### Design

Given its multidimensional nature, ethical leadership is well suited to be investigated through an integrative literature review, which allows for synthesis across diverse study designs and contexts.^17^ This approach supports the advancement of evidence-based nursing knowledge and helps identify both the current understanding and areas needing further exploration.^18^

This integrative literature review was conducted to systematically identify and synthesize existing literature on the prerequisites for ethical leadership in health and social care contexts, particularly from the perspective of health and social care leaders. The review was reported according to the Preferred Reporting Items for Systematic Reviews and Meta-Analyses (PRISMA) 2020 guidelines^19^ to ensure transparency and rigor. The protocol for this review was registered in the International Prospective Register of Systematic Reviews (PROSPERO, CRD420250656443). Preliminary data synthesis was based on inductive content analysis, following a modified structured five-phase approach described by Bingham.^20^

### Search methods

A comprehensive literature search was undertaken by the first author. The search was conducted in six electronic databases: PubMed, CINAHL, Cochrane Library, Medic, Business Source Ultimate, and Sociology Source Ultimate. A preliminary search was conducted on January 19, 2025 and updated on February 20, 2025. No time restrictions were applied to allow for comprehensive coverage. Additionally, a manual reference list screening was performed to identify relevant studies missed in the database searches. A library information specialist assisted in developing the search strategy.

Boolean operators, truncation, and controlled vocabulary (e.g., MeSH terms) were used to optimize sensitivity and specificity. The final search terms included combinations such as “Ethical Leadership” AND (lead* OR leadership OR management OR “Leader Perspective”), AND (nurse OR nursing OR “Healthcare Leadership” OR “Healthcare Executives” OR “Health Services Administration” OR “Healthcare Professionals”) AND (“Health Services” OR “Social Services” OR “Healthcare Organizations” OR “Healthcare System” OR “Social Care” OR organization). The word “prerequisites” was intentionally omitted from the search terms to avoid narrowing the scope. In the Finnish-language Medic database, which was the only Finnish-language database used for this review, the search terms “eetti* AND johta*” were applied. These terms were selected to capture various forms of ethical leadership terminology in Finnish (e.g., *eettinen johtaminen*, *eettinen johtaja*, and *eettinen johtajuus*), recognizing that leadership roles in Finnish health and social care may carry different titles. Despite the variety of roles, the concept of leadership is essentially shared across these positions. A full search strategy for each database is presented in Table 1.

**Table 1.** Search strategies, databases, and number of records retrieved.

Database	Search	Search phrase	Records retrieved
Business Source ultimate	#1	(ethic* AND (leader* OR manag*)	62,717
	#2	(lead* OR leadership OR management OR “Leader Perspective”) AND (nurse OR nursing OR “Healthcare Leadership” OR “Healthcare Executives” OR “Health Services Administration” OR “Healthcare Professionals”) AND (“Health Services” OR “Social Services” OR “Healthcare Organizations” OR “Healthcare System” OR hospital OR organization)	21,326
	FINAL SEARCH #1 AND #2	(“Ethical Leadership”) AND (lead* OR leadership OR management OR “Leader Perspective”) AND (nurse OR nursing OR “Healthcare Leadership” OR “Healthcare Executives” OR “Health Services Administration” OR “Healthcare Professionals”) AND (“Health Services” OR “Social Services” OR “Healthcare Organizations” OR “Healthcare System” OR hospital OR organization)	24
CINAHL	#1	(Ethic* AND lead*) OR (Ethic* AND manag*)	17,739
	#2	(nurs* OR nurses OR nursing) AND (“Health*” OR “Social*” OR organizat*)	331,681
	FINAL SEARCH #1 AND #2	(“Ethical Leadership”) AND (lead* OR leadership OR management OR “Leader Perspective”) AND (nurs* OR nurses OR nursing) AND (“Health*” OR “Social*” OR organizat*)	85
Cochrane library title/abstract/keyword	#1	(ethic* NEXT leader* OR ethic NEXT *manag*) AND (organizati* OR health* NEXT care OR social NEXT care)	1326
	FINAL SEARCH #2	(ethic* NEXT leader* OR ethic *manag*) AND (organizati* OR health* NEXT care OR social NEXT care) AND leadership*	17
PubMed	#1	(lead* OR leadership OR management) AND (ethic* OR “Ethical Leadership”)	104,896
	#2	(lead* OR leadership OR management) AND (ethic* OR “Ethical Leadership”) AND (health services OR organizat*)	46,548
	FINAL SEARCH #1 AND #2	“Ethical Leadership”) AND (lead* OR leadership OR management OR “Leader Perspective”) AND (nurse OR nursing) AND (“Health Services” OR “Social Services” OR organizat*)	38
Sociology source ultimate	#1	(“Ethic* AND Leader*”) OR (“Ethic* AND Manage*”)	149
	FINAL SEARCH #2	((“Ethic* AND Leader*”) OR (“Ethic* AND Manage*”)) AND (organiza* OR healthcare OR “social care” OR “health services” OR “social services”) AND (“Ethical Leadership” OR “Ethical Management”)	7
Medic	FINAL SEARCH	Eetti* AND johta*	12

### Study selection and data extraction

Studies were eligible if they (1) were empirical studies on ethical leadership that addressed the prerequisites for ethical leadership; (2) examined these prerequisites from the perspective of leaders, managers, or supervisors; (3) were conducted in healthcare, social care, or integrated service settings; and (4) were published in English or Finnish. Studies were excluded if they (1) did not address the prerequisites for ethical leadership from the perspective of leaders (“Irrelevant focus/Not addressing ethical leadership prerequisites” in the PRISMA diagram); (2) lacked empirical data (e.g., opinion pieces or editorials); or (3) presented only the perspectives of patients, students, or employees without incorporating leadership viewpoints.

Two reviewers independently conducted title and abstract screening using Covidence (https://www.covidence.org), a web-based screening and data extraction tool recommended by the Cochrane Collaboration.^21^ Full-text articles were then assessed for eligibility according to the inclusion criteria. Disagreements were resolved through discussion, with an input from a third reviewer when necessary. Reasons for exclusion were recorded to ensure transparency. A structured data extraction form was used to systematically collect relevant information from each included study. Extracted data included author(s), publication year, country, study aim, design, setting, participant characteristics, data collection, and analysis methods reported prerequisites for ethical leadership, influencing factors, and any applied theoretical frameworks (Table 2). A second reviewer verified the extracted data, and discrepancies were resolved through a team consensus.

**Table 2.** Overview of the included studies (*n* = 9) and their methodological quality assessed using the mixed methods appraisal tool (MMAT) (Hong et al., 2018).

Study	Purpose of the study	Sample	Data collection methods	Analysis methods	MMAT score (0–5)
Barkhordari-Sharifabad et al. (2017)	To analyze the outcomes and organizational impact of ethical leadership in nursing practice	Nursing leaders and managers, *n* = 18	Semi-structured interviews	Qualitative content analysis	4
Barkhordari-Sharifabad et al. (2018)	To examine how ethical leadership contributes to nurses’ professional growth and development	Primarily nurse managers and faculty members with leadership responsibilities, *n* = 14	Semi-structured interviews	Content analysis and constant comparison	5
Denier et al. (2019)	To explore values-based leadership as a form of ethical leadership in healthcare management, and how it shapes organizational culture	Healthcare managers *n* = 15	Qualitative interviews	Grounded theory	5
Sinkkonen & Laulainen (2010)	To examine how municipal social sector leaders perceive their ability to act according to ethical principles in their leadership roles and explore ethical dilemmas they encounter	Social service leaders *n* = 209	Structured questionnaire (numeric scales, open-ended questions)	Factor analysis and qualitative content analysis	4
Makaroff et al. (2014)	Explore ethical leadership in nursing through meta-ethnography	Participants, drawn from four qualitative studies, inc. nurse leaders *n* = 601	Meta-ethnography	Lines-of-argument synthesis	4
Morin & Talbot (2023)	To investigate the effects of ethical leadership on healthcare management in the Nunavik region, with attention to organizational and systemic barriers	Healthcare managers *n* = 17	Qualitative interviews	Retrospective case study analysis	5
Storaker et al. (2022)	To understand the ethical challenges faced by nurse leaders in somatic hospital settings and how these challenges influence ethical leadership	Nurse leaders, *n* = 10	Hermeneutical interviews	Hermeneutical analysis	5
Trong Tuan (2012)	To examine how clinical governance initiatives influence leadership practices, particularly in promoting ethical leadership and trust within healthcare settings	CEO, head doctors, head nurses; primarily individuals in leadership positions, *n* = 51	Document analysis, field observations, in-depth interviews	Thematic analysis	5
Yu et al. (2025)	To investigate how the ethical climate within hospitals influences moral resilience, ethical competence, and the development of ethical leadership among head nurses	Head nurses, *n* = 309	Survey	Pearson’s correlation and structural equation modeling	5

### Quality appraisal and data extraction

The methodological quality of the included studies was assessed using the Mixed Methods Appraisal Tool (MMAT).^22^ Two reviewers independently evaluated each study based on five criteria, with a maximum possible score of five. Any discrepancies in scoring were discussed and resolved by consensus. No studies were excluded due to quality concerns; all included studies received scores of either 4 or 5, ensuring that methodologically robust studies were synthesized. Relevant data were extracted into a structured summary table, which includes information on the author(s), year, country, study aim, design, sample, data collection methods, key findings, and methodological quality (MMAT) (Table 2).

### Data synthesis and analysis

The data analysis was conducted using a modified version of Bingham’s^20^ five-phase model, applied inductively throughout all phases. While Bingham’s original model integrates inductive and deductive elements in the fifth phase, this review focused on inductive category formation based on the content of the included studies. First, all included studies were read thoroughly to identify content relevant to the research question. In the second phase, meaningful units—based on clearly identifiable expressions in the text—were condensed and summarized at the manifest level to enhance interpretability. The analysis remained inductive, and no interpretations beyond the original content were made. Third, open coding was used to generate initial codes that reflected recurring ideas or patterns in the data. These codes were then clustered into preliminary categories based on conceptual similarities. The analysis resulted in five main categories, each comprising three to five sub-categories, totaling 18 sub-categories altogether. Finally, the categories were contrasted with existing literature to identify novel insights and contextual differences in how the prerequisites for ethical leadership were conceptualized.

The process was iterative, and two researchers independently conducted the coding and categorization. Any differences were discussed and resolved collaboratively. Peer debriefing and thorough documentation throughout the process enhanced methodological rigor. In line with integrative review methodology, findings from quantitative studies were qualitatively transformed and synthesized using a data-based convergent approach.^22,23^ Contradictions and variations across studies were also considered in light of contextual factors and research designs. This analytic strategy allowed for the integration of diverse perspectives while maintaining the contextual and conceptual richness of the original findings. Examples of the analytical process, from meaning units to main categories, are presented in Table 3.

**Table 3.** Examples of the inductive data analysis process based on Bingham’s modified five-phase model (Bingham et al. 2023).

Meaning unit/familiarization	Condensed meaning unit/meaning condensation	Open coding (initial code)	Category development (sub-category)	Main category/integration
“I wanted to respect my values. For me, it was to ensure that there was a service that was transparent and ethical.” (Morin & Talbot, 2023, p. 591)	Stands by values despite unethical leadership; resists pressure; defends ethical practice	Upholding ethical values under pressure; resisting unethical leadership	Moral resilience	Leader-centric prerequisites
“Ethical leadership is the day-to-day expression of one’s commitment to other persons and the ways in which human beings relate to one another in their daily interactions.” (Makaroff et al., 2014, p. 651)	Ethics is lived through everyday interpersonal interactions	Ethics embodied in daily behavior	Ethical commitment	
“Ethical leaders can influence their personnel by demonstrating honest and ethical behavior. Through this, they would lead the nurses toward ethics.” (Barkhordari-Sharifabad et al., 2018, p. 1057)	Leaders influence staff by acting honestly and ethically	Leading by ethical example	Consistency and modeling	Ethical organizational culture
“This also illustrates that creating a values-based organizational culture is not an individual matter, to be done by the manager on one’s own. It has to be done, together with everyone involved.” (Denier et al., 2019, p. 7)	Creating a values-based culture is a collective effort involving everyone	Building shared ethical culture collaboratively	Shared values	
“A clinical governance initiative, when effectively implemented, can function as a lever for behavioural transformations in the hospital towards ethical leadership.” (Trong Tuan, 2012, p. 224)	Clinical governance supports ethical leadership through structural change	Governance structure enabling ethical behavior	Supportive structures and infrastructures	Resource sufficiency
“Such support and guidance should be found in Codes of Ethics, Professional Standards of Practice, organizational codes of ethics, and resources for ethical practice that focus on the development of ethical leadership.” (Makaroff et al., 2014, p. 655)	Ethical leadership requires guidance through ethical codes, standards, and resources	Need for clear ethical guidelines	Availability of ethical codes and standards	Ethical guidelines

## Findings

**Figure 1.**
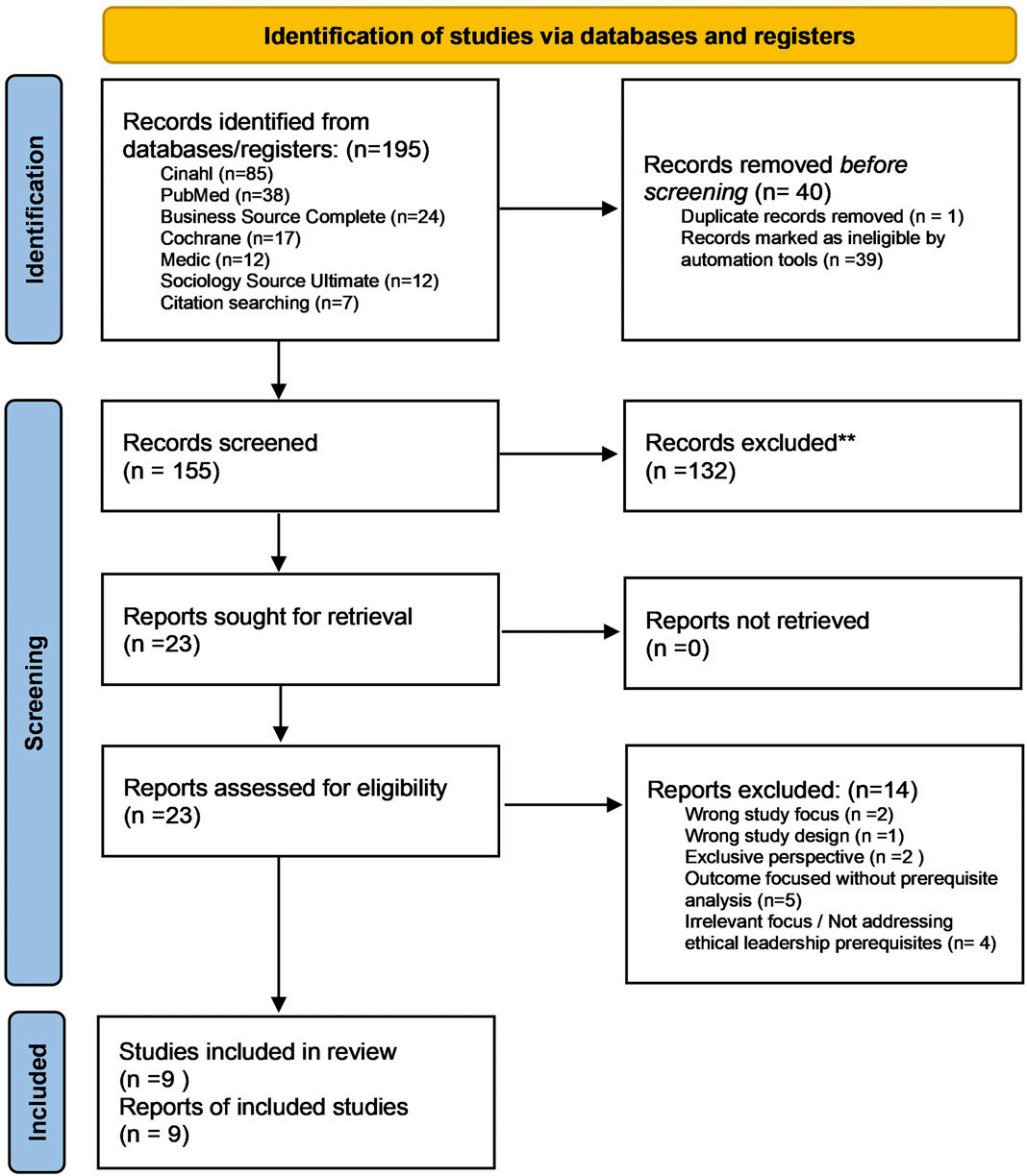
PRISMA flowchart illustrating the literature selection process. (Adapted from Page et al., 2021).

The initial database search yielded 195 records. After removing one duplicate manually and 39 duplicates identified by Covidence, 155 records were screened by their title and abstract. Of these, 132 were excluded for not meeting the inclusion criteria. A total of 23 full-text articles were assessed for eligibility, and 14 studies were excluded for the following reasons: outcome-focused without prerequisite analysis (*n* = 5), irrelevant focus or not addressing ethical leadership prerequisites (*n* = 4), exclusive perspective (*n* = 2), wrong study focus (*n* = 2), and wrong study design (*n* = 1). No ongoing or awaiting classification studies were identified. Consequently, nine studies were included in the final synthesis. A PRISMA flow diagram summarizing the selection process is presented in Figure 1.

### Characteristics of the studies retrieved

This review included nine peer-reviewed studies published between 2010 and 2025. The studies were conducted in various countries, including Canada (2 studies:^24,25^), Iran (2 studies:^26,27^), Finland,^28^ Belgium,^29^ China,^30^ Norway,^31^ and Vietnam.^32^ Most studies employed a qualitative research design (*n* = 7), typically descriptive or exploratory in nature. One study used a quantitative cross-sectional design,^30^ and one applied a mixed methods design combining qualitative interviews with document analysis.^25^

The study participants included mid- and senior-level healthcare leaders, such as nurse managers, hospital administrators, and executive-level decision-makers. The studies examined various aspects of ethical leadership, including its core components, implementation challenges, and support structures, including moral resilience,^31^ ethical competence,^26^ and organizational climate.^29,30^ Several studies also explored leaders’ perspectives on ethical principles,^32^ barriers to ethical practice,^24^ ethical challenges, and the role of organizational support in promoting ethical leadership.^28^

The studies utilized a variety of theoretical and analytical frameworks, reflecting the interdisciplinary nature of ethical leadership research. These frameworks included ethical leadership theory,^24^ organizational ethics,^29^ clinical governance,^32^ and values-based leadership models.^25^ Methodologically, the studies employed diverse qualitative approaches such as content analysis,^26^ meta-ethnography,^24^ hermeneutic interpretation,^31^ and grounded theory.^28^ This variety of perspectives and analytical strategies contributed to a multidimensional understanding of the prerequisites for ethical leadership in complex health and social care environments.

### Prerequisites of ethical leadership

**Figure 2.**
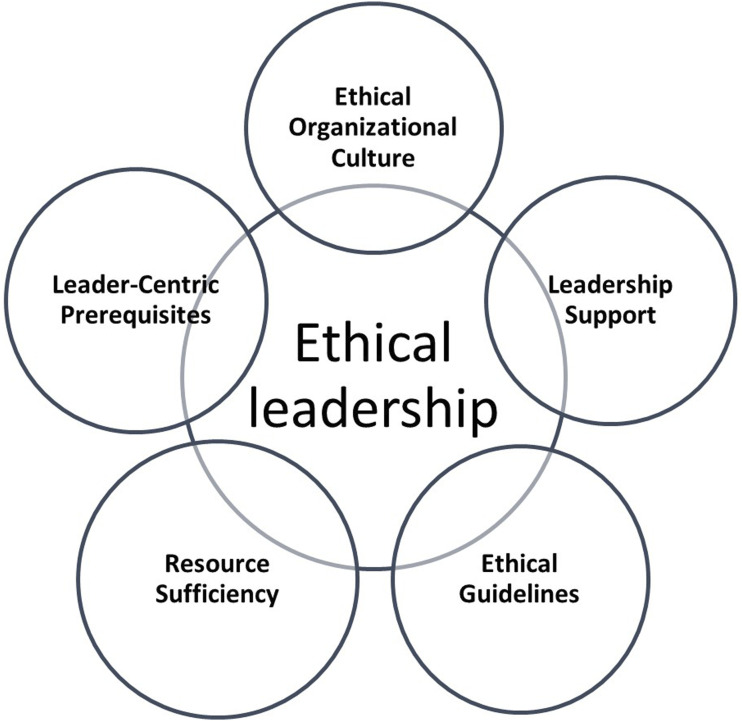
Synthesis of the five categories of prerequisites for ethical leadership in health and social care.

Five categories were synthesized from the included studies to describe the prerequisites of ethical leadership in health and social care from the perspective of leaders: (1) leader-centric prerequisites, (2) ethical organizational culture, (3) leadership support, (4) ethical guidelines, and (5) resource sufficiency (Table 4, Figure 2).

**Table 4.** Key prerequisites for ethical leadership in social and health care organizations.

Category	Description	Verified references in text
1. Leader-centric prerequisites	Personal attributes and competencies such as moral sensitivity, ethical awareness, resilience, fairness, and ability to express ethical concerns. These traits enable leaders to make justifiable decisions and model ethical behavior	Barkhordari-Sharifabad et al. 2018; Denier et al. 2019; Makaroff et al. 2014; Morin & Talbot 2023; Sinkkonen & Laulainen 2010; Storaker et al. 2022; Yu et al. 2025
2. Ethical organizational culture	A working environment where ethical values are visible, shared, and actively promoted through policies, norms, and leadership practices. An ethical culture strengthens coherence and accountability	Barkhordari-Sharifabad et al. 2017; Barkhordari-Sharifabad et al. 2018; Denier et al. 2019; Makaroff et al. 2014; Morin & Talbot 2023; Sinkkonen & Laulainen 2010; Storaker et al. 2022; Trong Tuan 2012; Yu et al. 2025
3. Leadership support	Institutional and supervisory structures such as mentoring, peer networks, and open communication that empower leaders to act ethically and feel valued in decision-making	Barkhordari-Sharifabad et al. 2017; Barkhordari-Sharifabad et al. 2018; Makaroff et al. 2014; Morin & Talbot 2023; Sinkkonen & Laulainen 2010; Storaker et al. 2022; Trong Tuan 2012; Yu et al. 2025
4. Ethical guidelines	Formalized ethical codes and policies that guide leaders in ethically complex situations and promote consistency in ethical reasoning and actions across all organizational levels	Barkhordari-Sharifabad et al. 2017; Barkhordari-Sharifabad et al. 2018; Denier et al. 2019; Makaroff et al. 2014; Morin & Talbot 2023; Sinkkonen & Laulainen 2010; Storaker et al. 2022; Trong Tuan 2012; Yu et al. 2025
5. Resource sufficiency	Adequate human, financial, and structural resources that support ethical leadership implementation and reduce ethical strain caused by under-resourcing or systemic limitations	Barkhordari-Sharifabad et al. 2017; Barkhordari-Sharifabad et al. 2018; Denier et al. 2019; Makaroff et al. 2014; Morin & Talbot 2023; Sinkkonen & Laulainen 2010; Storaker et al. 2022; Trong Tuan 2012; Yu et al. 2025

#### Leader-centric prerequisites

Leader-centric prerequisites as a category was further sorted to comprise five sub-categories: ethical awareness and moral sensitivity,^24,27,28,31^ ethical commitment,^27–30^ ethical competence,^25,27,28,30,31^ moral resilience,^25,26,30^ and ethical communication skills.^24,26,29,31^ The reviewed studies revealed that, together, these personal attributes formed the foundation for ethical leadership by enabling leaders to recognize and respond to ethical issues in complex care environments. Ethical awareness and moral sensitivity were particularly important in identifying moral dimensions that might otherwise remain unnoticed in daily operations.^25,27–31^

Ethical commitment reflected a leader’s deep-rooted dedication to moral values and the courage to act accordingly, forming a foundation for sustained ethical leadership.^27,28^ Ethical competence referred to the ability to identify ethical dilemmas, make justifiable decisions, and uphold integrity in the face of conflicting demands.^24,29–31^ Moral resilience indicated the capacity for leaders to maintain ethical integrity under organizational stress or uncertainty.^26,30^ Finally, ethical communication skills enabled leaders to clearly express ethical concerns and promote a shared understanding of ethical expectations throughout the organization.^31^

These personal competencies were found to interact with organizational factors as leaders with strong ethical competence could more effectively utilize support structures and contribute to developing ethical organizational cultures.^27–29^ However, even leaders with strong ethical commitment faced challenges when organizational support was lacking, suggesting that personal qualities alone were necessary but insufficient for sustainable ethical leadership.^25,31^

#### Ethical organizational culture

Ethical organizational culture as a category comprised four sub-categories: shared values,^26–29,31,32^ ethical climate,^24,26,27,29,31,32^ consistency and modeling,^24,27,29,31^ and openness to ethical concerns.^26,29,31^ These elements described how organizational values, climate, communication, and leadership behaviors shaped the conditions for ethical leadership. An ethical organizational culture provided a broader context in which ethical leadership could thrive. Health and social care institutions with clearly defined ethical frameworks, explicit expectations, and structured accountability mechanisms fostered environments conducive to ethical leadership.^29,30^ A systematically developed ethical culture integrated core values into organizational structures and professional practices.^28^

Ethical leadership and organizational culture were found to demonstrate a reciprocal relationship where each reinforced the other. Multiple studies described this interdependence, noting that neither could fully thrive in isolation.^24–26,28,29,32^ A strong ethical climate supported the consistent implementation of ethical leadership and fostered professional growth and organizational unity.^26^

Among the studies, it was found that organizations that had embedded ethics-based policies and leadership training had cultivated environments where ethical standards were consistently upheld. Leaders supported these cultures by modeling integrity, reducing uncertainty, and promoting consistency in decision-making processes.^27,28,31^ The integration of ethics education into operational structures strengthened the ethical awareness and decision-making competence of the leaders.^28,30,31^

The data revealed that ethical leadership development was hindered when cultures were characterized by avoidance or organizational silence regarding ethical issues. Similarly, inconsistent organizational messaging and the absence of a shared ethical language presented barriers to developing a robust ethical culture that could support leadership.^31^ A disjointed organizational culture, unclear authority, and lack of ethical reinforcement also weakened the ability to maintain ethical integrity and consistency in leadership practice.^27^

#### Leadership support

The category of leadership support included four sub-categories that described key support mechanisms for ethical leadership: guidance and mentoring relationships,^25–27,30,31^ inclusion in decision-making,^25,28,29^ top-level collaboration,^25,29,32^ and access to ethical infrastructure.^24,26,29,30,32^ These elements highlighted how organizational and peer-level support enabled leaders to integrate ethical principles into daily practice and decision-making. In the studies, organizational and supervisory support emerged as a critical prerequisite for ethical leadership in health and social care settings. Leaders consistently reported that mentoring, encouragement, and systemic backing were significant for integrating ethical principles into everyday leadership practices.^24,25,32^ Organizational alignment with ethical values and established ethical structures enabled leaders to fulfill their ethical responsibilities effectively.^24,30^

The quality of relationships between senior management and health and social care leaders was found to have significantly impacted both ethical leadership and overall organizational performance.^27^ Strengthening ethical leadership identity occurred through reciprocal processes between individual leaders and organizational systems.^25^ Supportive ethical environments were characterized by systematic approaches to assessing moral dimensions, including structures such as ethics committees, peer support networks, and ethics training.^28,30–32^

The studies highlighted significant barriers to ethical leadership when support was lacking. Leaders described experiencing responsibility for implementing decisions without having real authority or inclusion in decision-making processes.^24–26^ Perceived gaps in values between middle and top management contributed to feelings of being unheard or excluded.^25,26^

Hierarchical dynamics in health and social care organizations were found to have significantly impacted ethical leadership development. Nurse leaders often experienced these hierarchies as feeling undervalued or professionally marginalized compared to medical leadership.^24,25^ Exclusion from ethically significant decisions undermined value-based leadership and created moral conflict.^25,26,28,31^

#### Ethical guidelines

Ethical Guidelines as a category comprises three sub-categories: the availability of ethical codes and standards,^24,26–32^ ethics education and training,^24,27,29–32^ and ethical consistency in decision-making.^24,27,29^ In the studies, ethical guidelines supported and directed ethical leadership practices by providing clear expectations and tools for navigating moral dilemmas. Established ethical codes offered direction for nurse leaders and reinforced ethical decision-making at all organizational levels.^26,29^ These guidelines served as reference points that ensured consistency and accountability in ethical practices.^28^

Organizational ethical guidelines were found to have established explicit expectations and provided important tools for ethical decision-making. Institutions that integrated structured ethical frameworks into leadership policies strengthened ethical leadership by fostering ethical reflection and professional development.^24,25,27^ Ethical codes helped balance factual considerations with moral values in complex decision-making situations.^29^

The data revealed that guidelines had to be actively implemented to be effective. Guidelines alone proved insufficient without integration into leadership training and organizational culture.^32^ Without proper implementation, ethical policies remained theoretical and failed to support nurse leaders in navigating complex ethical dilemmas.^27^ Institutions that provided continuous ethics training and consistently reinforced ethical expectations empowered leaders to apply ethical principles effectively.^30,31^

Ethical guidelines served multiple functions in supporting ethical leadership: they bridged individual moral perspectives with broader leadership expectations and legal requirements^28^; contributed to moral resilience and ethical competence when integrated into the broader ethical climate^30^; and offered clarity in ethical dilemmas, reducing uncertainty and moral distress among nurse leaders.^24,31^ Well-defined guidelines also helped maintain ethical leadership integrity when faced with external pressures such as financial constraints or hierarchical challenges.^26^

#### Resource sufficiency

The category of resource sufficiency comprised two sub-categories: adequate staffing and time^25,26,28,29,31^ and supportive structures and infrastructure.^24,26,29,30,32^ Adequate financial, human, and structural resources constitute a fundamental prerequisite for ethical leadership in health and social care settings. In the studies, leaders frequently encountered ethical tensions due to insufficient resources, such as understaffing, time pressure, or inadequate institutional support, all of which significantly hindered ethical decision-making.^25,26,28,29^ A stable and well-managed resource base enabled leaders to maintain ethical standards, ensure continuity of care, and manage ethically complex situations without compromising core values.^26,29^ Having adequate staffing and time was found to directly affect a leader’s capacity to act ethically and make principled decisions under pressure.^25,26,28,29^ Supportive structures and infrastructure were also found to significantly determine the extent to which leaders can handle stressful situations ethically.^26,29^

The research evidence consistently demonstrated the consequences of resource scarcity on ethical leadership. Limited budgets and staff shortages forced nurse leaders to make difficult compromises, thereby undermining consistent ethical leadership.^27,31^ Structural deficiencies, high staff turnover, and long-standing vacancies also directly weakened the ability of leaders to act in accordance with their moral obligations.^25^ Although moral resilience was an important personal resource, it could not replace structural support. Ethical competence diminished in contexts where organizational support and staffing were inadequate, leading to ethical distress despite individual moral resources.^30^

Resource sufficiency was also found to intersect significantly with other prerequisites for ethical leadership. Well-resourced organizations enabled ethical leadership by embedding ethics into budgeting, leadership training, and daily operations. Clinical governance initiatives succeeded in promoting ethical practices only when they were adequately resourced. Ethics was viewed not merely as a matter of policy but as a practice supported by concrete resource decisions. Furthermore, resources directly affected leadership support systems and organizational culture, as they formed the enabling environment in which ethical leadership could flourish.^32^

Challenges in achieving resource sufficiency were found to include competing priorities in budget allocation, difficulties in staff recruitment and retention, and the challenge of justifying investments in ethical infrastructure amid financial constraints. Organizations often struggled to balance immediate service demands with long-term investments in ethical leadership. Moreover, unstable or insufficient support structures caused ethical initiatives to remain fragile or short-lived, undermining their impact and sustainability over time.^25,26,31^ When these challenges went unaddressed, ethical leadership became reactive, vulnerable to external pressures,^25,31^ and detached from its values-based foundation.^25,26^

## Discussion

This integrative review synthesized current research on the prerequisites for ethical leadership from the perspective of health and social care leaders. Despite the small number of included studies, this limited evidence highlights the novelty and research gap in the field. The findings demonstrate that ethical leadership is not merely a function of individual competencies, but a systemic process shaped by organizational structures, culture, and available resources.^28–30^ This is supported by earlier reviews that have emphasized how ethical leadership is context-sensitive and shaped by both personal competencies and organizational environments.^3,33^ Five interrelated prerequisites were identified: leader-centric prerequisites, leadership support, ethical organizational culture, ethical guidelines, and resource sufficiency.

While personal characteristics and competencies, such as ethical sensitivity, value awareness, and moral resilience, are important,^25,27,31^ they remain insufficient without a supportive organizational context.^30^ Prior research has similarly found that leaders’ agreeableness, conscientiousness, and moral identity positively shape perceptions of ethical leadership, particularly when leaders are supported by systems that enable reflection and role modeling.^33^ Therefore, individual-level competencies must be embedded within a broader framework of leadership support and structural enablers. This review thus advocates shifting from an individual-centered view to a broader organizational and structural perspective.^28,29^

Leadership support emerged as a consistent theme across the studies. Receiving support from colleagues or leaders, mentoring, training, and ethically grounded leadership development programs had proved to be critical enablers of ethical practice.^24,25^ Broader literature supports this by demonstrating that ethically anchored leadership development, such as structured mentoring and training that emphasize fairness, transparency, and moral responsibility, can equip leaders to manage ethical complexity and build inclusive, value-driven workplaces.^3,15^ These forms of support do not operate in isolation but interact with personal competencies and organizational culture to facilitate ethical decision-making. Trustful relationships between different leadership levels enhances ethical action, whereas a lack of support or a value misalignment can significantly hinder it.^26,31^

In addition to leadership support, clearly communicated and practically integrated ethical guidelines that reflect an organization’s underlying normative ethical principles play a crucial role in guiding the ethical decision-making and actions of leaders. When integrated into daily ethical leadership practices and supported by education and accountability mechanisms, ethical guidelines promote clarity and consistency.^28–30^ However, their symbolic value remains limited without active implementation and ongoing reinforcement throughout the organization.^24,32^ The ethical climate of an organization, including how guidelines are interpreted and embedded in practice, was identified as a pivotal factor in maintaining ethical standards and promoting employee engagement.^11^

Resource sufficiency emerged as a foundational condition underlying all aspects of ethical leadership.^25,27^ This encompasses adequate staffing levels and stability, sufficient financial resources for service provision, appropriate infrastructure, time allocated for ethical reflection, and access to professional development.^29,32^ These findings echo previous evidence showing that, without structural and financial capacity, even highly ethical leaders may be unable to act ethically.^34^ Staffing shortages, financial pressures, and time constraints were consistently found to weaken the ability of leaders to act ethically, while adequate resources were seen to enable ethics training, supportive infrastructures, and the development of robust ethical organizational cultures.^30,31^

The findings highlight that organizational culture functions as a critical prerequisite for ethical leadership. Transparency, shared values, and open dialogue within the organization foster ethical decision-making and leadership behaviors,^25,27^ whereas organizational silence and inconsistency can undermine these conditions and weaken leaders’ capacity for ethical action.^28,31^ Practical examples from healthcare organizations implementing ethical leadership frameworks suggest that consistent value alignment, leader visibility, and psychological safety are vital cultural components.^11,35^ Ethical leadership does not operate in a vacuum; it relies on structural support, leadership development, a strong ethical culture, and adequate resources. Recognizing these factors as interdependent enablers helps shift the focus from individual competencies to systemic conditions that sustain ethical practice. These insights provide actionable guidance for leadership development, governance structures, and policy formation in healthcare systems.^25,29^

In future research, scholars should explore how these prerequisites interact across different cultural and health and social care settings and develop practical tools to assess their presence and strength.^30,33^ In particular, Singh and Vashist^3^ recommend the development of assessment tools that include dimensions such as sustainability, empowerment, and power-sharing. Broadening the focus beyond nursing leadership to integrated health and social care services will further enhance the applicability and impact of ethical leadership frameworks in practice. Cross-sectoral and cross-cultural comparisons may also help uncover how ethical leadership is shaped by institutional logic and policy environments.

## Methodological considerations and limitations

This review has several methodological and conceptual limitations. First, most of the included studies originated from nursing science, which may limit the generalizability of the findings to other types of leadership roles within health and social care settings. However, considering that nurses represent the largest professional group in healthcare, the nursing perspective provides valuable insights into ethical leadership in healthcare organizations. Second, ethical leadership was not consistently or systematically defined across the included studies. While it was broadly approached in terms of both individual competencies and organizational processes, this review did not analyze in detail how each study conceptualized ethical leadership. This lack of definitional clarity may have influenced the identification and interpretation of prerequisites, which highlights the need for future research to apply more coherent, theory-informed definitions for how prerequisites are interpreted. This also underscores the need for more consistent, theory-based frameworks in future research. Moreover, this review only included studies published in English and Finnish, which may have excluded relevant research published in other languages.

One conceptual limitation of this review concerns the potential overlap and ambiguity between the terms *prerequisites*, *enablers*, and *conditions*. While all included factors were interpreted as *prerequisites* due to their consistent association with ethical leadership, the literature does not always clearly differentiate between what is required in ethical leadership, what facilitates it, and what sustains it.

The identification of prerequisites is inherently subjective and context-dependent. In public sector leadership, prerequisites are not universally present but rather reflect varying combinations of contextual demands and available resources, which may or may not support leadership and staff wellbeing.^8^ Future reviews could address this limitation by clarifying how such concepts are defined and applied, thereby improving conceptual precision and analytical consistency.

The relatively small number of studies reflects both the specificity of the inclusion criteria and the scarcity of focused research. While inductive analysis allowed conceptual depth, it also involved interpretative reasoning, which may have introduced subjectivity. This was mitigated by rigorous selection criteria, quality appraisal, and the use of multiple reviewers.

Although the findings offer a conceptual foundation for understanding the prerequisites for ethical leadership, their applicability across leadership levels, service areas, and cultural contexts remains limited. Future studies should investigate how these components function in various systems—potentially as enabling conditions rather than strict prerequisites—and develop tools to assess their presence and interaction.

Identifying these prerequisites is a key step for designing evidence-based leadership development programs, strengthening ethical infrastructures, and ensuring that ethical leadership is not left to individual moral effort alone. A clear understanding of the conditions that support ethical leadership can inform targeted interventions and systemic changes within healthcare organizations. Despite these limitations, this review advances the field by providing a structured synthesis of the prerequisites for ethical leadership, and it highlights directions for future research across global health and social care settings.

## Conclusion

This review underscores that ethical leadership is not solely grounded in individual competencies; it requires structured organizational prerequisites to be realized and sustained in practice. The findings identify five core prerequisites that function as key enablers of ethical leadership and must be actively implemented and maintained for it to be sustainable in health and social care contexts. While ethical leadership is often associated with positive values, the results reveal a complex synthesis shaped by competing demands, systemic constraints, and organizational dynamics. No single ethical tool or leadership style is sufficient to address the wide range of ethical dilemmas encountered in practice. Instead, ethical leadership requires the ability to flexibly apply context-appropriate leadership approaches, supported by integrated ethical frameworks, organizational structures, and institutional commitment.

Ethical leadership should not be viewed as static competence but as a dynamic and evolving process embedded in all aspects of organizational decision-making. It demands the capacity to balance conflicting values, make difficult decisions, and maintain ethical integrity in evolving and often constrained environments.

These findings offer practical implications for health and social care organizations. Leadership structures should incorporate ethics committees, mentorship programs, and dedicated resources to support ethical practices. At the policy level, integrating ethical leadership development into governance models and accountability systems can further institutionalize ethical decision-making.

As health and social care environments become increasingly complex, ethical leadership must remain adaptable, reflective, and systemically supported. Organizations that embed ethical leadership as an integral and strategic part of their culture and operations are better positioned to promote integrity, accountability, and the quality of care across all levels of service.

In addition to organizational and policy-level strategies, leaders can also take concrete actions to strengthen ethical leadership in their daily practice. These include engaging in regular ethical reflection and dialogue with staff, integrating ethical values into day-to-day decision-making, advocating for transparency and fairness, and addressing ethical concerns proactively. Participating in leadership training focused on ethical decision-making and building supportive peer networks may further equip leaders to model and sustain ethical conduct, particularly in ethically demanding or resource-constrained environments.

## Data Availability

No datasets were generated or analyzed during the current study. Not applicable.
